# Beyond the “3 Ps”: A critical appraisal of the non-endocrine manifestations of multiple endocrine neoplasia type 1

**DOI:** 10.3389/fendo.2022.1029041

**Published:** 2022-10-17

**Authors:** Steven G. Waguespack

**Affiliations:** Department of Endocrine Neoplasia and Hormonal Disorders and the Children’s Cancer Hospital, The University of Texas MD Anderson Cancer Center, Houston, TX, United States

**Keywords:** MEN1, angiofibroma, collagenoma, meningioma, ependymoma, lipoma, leiomyoma, breast cancer

## Abstract

Multiple endocrine neoplasia type 1 (MEN1), an autosomal-dominantly inherited tumor syndrome, is classically defined by tumors arising from the “3 Ps”: Parathyroids, Pituitary, and the endocrine Pancreas. From its earliest descriptions, MEN1 has been associated with other endocrine and non-endocrine neoplastic manifestations. High quality evidence supports a direct association between pathogenic *MEN1* variants and neoplasms of the skin (angiofibromas and collagenomas), adipose tissue (lipomas and hibernomas), and smooth muscle (leiomyomas). Although CNS tumors, melanoma, and, most recently, breast cancer have been reported as MEN1 clinical manifestations, the published evidence to date is not yet sufficient to establish causality. Well-designed, multicenter prospective studies will help us to understand better the relationship of these tumors to MEN1, in addition to verifying the true prevalence and penetrance of the well-documented neoplastic associations. Nevertheless, patients affected by MEN1 should be aware of these non-endocrine manifestations, and providers should be encouraged always to think beyond the “3 Ps” when treating an MEN1 patient.

## Introduction

Multiple endocrine neoplasia type 1 (MEN1) is a rare, autosomal-dominantly inherited tumor syndrome defined clinically by glandular hyperplasia and benign or malignant neoplasms involving two or more endocrine glands in a single individual (1, 2). It is caused by inactivating pathogenic DNA variants in the *MEN1* gene, located on chromosome 11q13, which functions as a tumor suppressor and encodes menin, a ubiquitous nuclear protein that plays a role in transcriptional regulation, genome stability, cell division and proliferation (3, 4). MEN1 represents an archetypal example of the "two hit" mechanism of disease, first described by Knudson in hereditary retinoblastoma (5). A heterozygous pathogenic germline *MEN1* variant is insufficient to induce tumor formation and thus a somatic chromosomal loss or loss-of-function mutation (the “second hit” affecting the wild type *MEN1* allele, thus causing biallelic loss) is required to cause disease (6–8). The “second hit” causes a loss of heterozygosity (LOH) at the *MEN1* locus in at-risk tissues and, in turn, decreased menin expression and attenuation of the ordinary constraints by menin on cell growth. LOH affecting the *MEN1* gene in a tumor is highly suggestive of causality but not always conclusive, given that genomic loss of 11q13 can also occur in sporadic neuroendocrine tumors (9–11) and also due to the possibility that deletions of other genes contiguous to *MEN1* on chromosome 11q13 may actually be responsible for a particular clinical phenotype.

Classically defined by tumors in the “3 Ps”: **P**arathyroids, **P**ituitary, and the endocrine **P**ancreas, other endocrine neoplastic manifestations include adrenocortical tumors, extra-pancreatic foregut neuroendocrine tumors, and very rarely pheochromocytoma. Even though MEN1 is considered an endocrine tumor syndrome, other neoplastic manifestations affecting the skin, adipose tissue, smooth muscle, central nervous system, and breast have been reported. The purpose of the current paper is to look beyond the 3 Ps and the other endocrine manifestations of MEN1 to provide a critical appraisal and state-of-the-art review of the often-overlooked, non-endocrine components of this fascinating hereditary syndrome.

## Methodology

Through July 2022, the author identified articles published in English through the U.S. National Library of Medicine (PubMed*^®^;* https://www.ncbi.nlm.nih.gov/pubmed) employing a systematic search using the following terms: “multiple endocrine neoplasia type 1”, “death”, “survival”, “series”, “breast cancer/carcinoma”, “dermatologic”, “skin”, “cutaneous”, “angiofibroma”, “collagenoma”, “lipoma”, “hibernoma”, “melanoma”, “leiomyoma”, “fibroid”, “ependymoma”, and “meningioma”. A secondary review of reference lists and subsequent manuscripts citing previously published papers led to the identification of additional relevant articles.

## Cutaneous tumors (angiofibromas and collagenomas)

**Figure 1:**
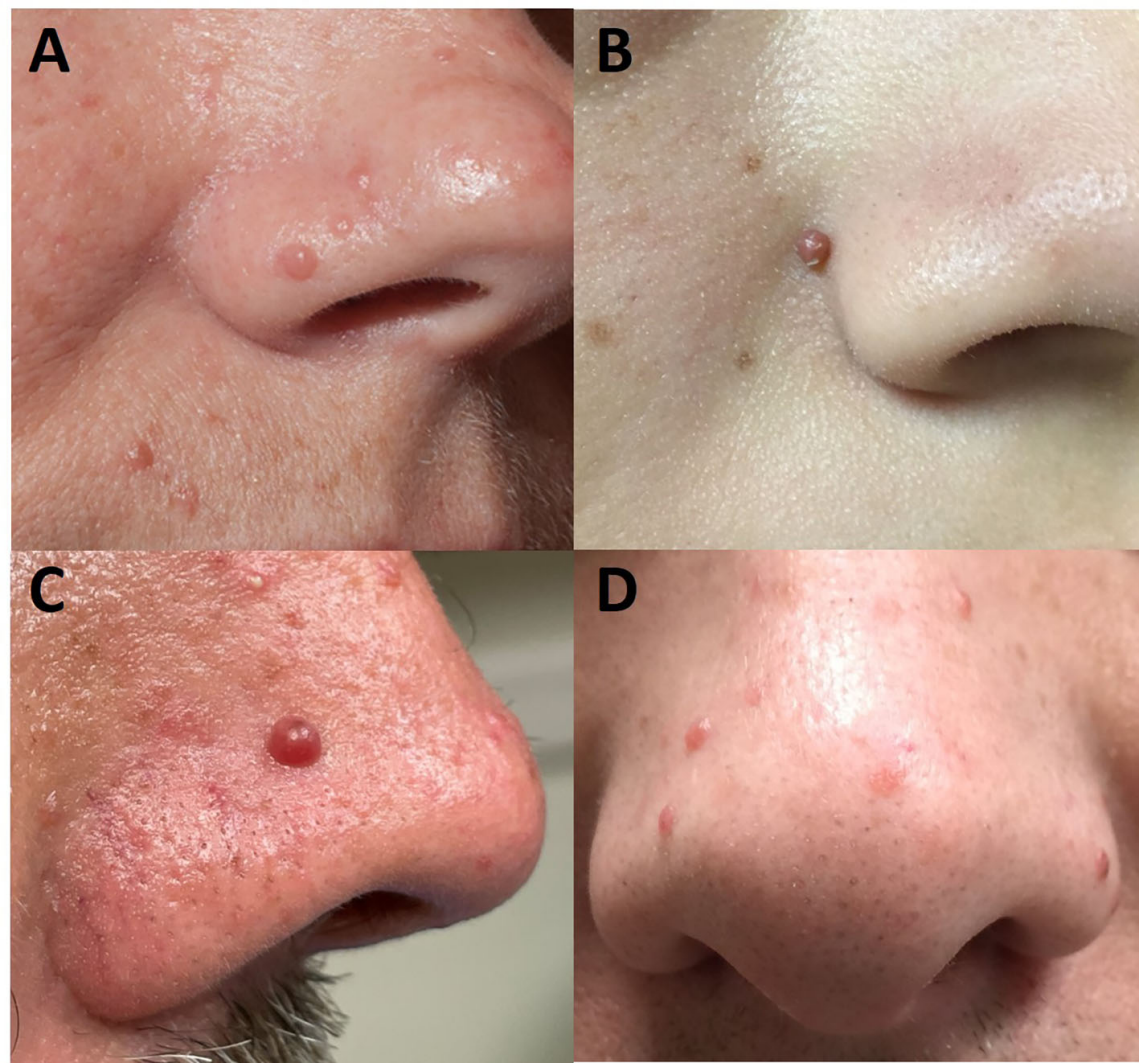
Facial angiofibromas as seen in a 39-year-old woman **(A)**, 14-year-old girl **(B)**, 48-year-old man **(C)**, and 43-year-old man **(D)** with multiple endocrine neoplasia type 1. Angiofibromas are telangiectatic, skin colored to pink to red-brown, dome-shaped papules that are histologically characterized by fibrous tissue and vascular proliferation. They are distributed primarily on the central part of the face, especially the nose.

MEN1 is one of several hereditary endocrine tumor syndromes associated with dermatologic manifestations (12). Long recognized but often unappreciated as a component of the clinical phenotype, the skin manifestations of MEN1 include angiofibromas and collagenomas (**Table 1** **;** **Figures 1** **-** **3**). Usually multiple and tending to be more commonly identified with increasing age in MEN1 patients (13–15), these dermatological manifestations can also be diagnosed in children (17–19) (**Figure 1**). In fact, they can be the earliest or only manifestation of MEN1 (13, 17, 20–23). Like other MEN1 manifestations, there is no clear genotype-phenotype correlation (24, 25). By age 40, the penetrance of angiofibromas and collagenomas in MEN1 patients has previously been estimated to be 85% and 70%, respectively (26). However, the true prevalence is difficult to ascertain due to the lack of large multi-institutional and prospective studies of MEN1 patients undergoing a thorough dermatologic examination. The first study that systematically examined unselected patients with MEN1 for skin manifestations was published in 1997 from the National Institutes of Health (NIH) (13). In 32 patients, angiofibromas and/or collagenomas were highly prevalent (29/32; 91%), including one patient in whom the clinical diagnosis of MEN1 was made before the biochemical confirmation of primary hyperparathyroidism (PHPT); no patients in a control group were identified to have multiple angiofibromas or collagenomas. A second study from the NIH was published in 2004 and included a systematic dermatologic examination in 110 consecutive patients with sporadic and MEN1-associated gastrinomas (15). In this study, 39/48 (81%) MEN1 patients had an angiofibroma or collagenoma, which were much more commonly seen in MEN1 patients compared with sporadic gastrinoma patients.

**Table 1:** Prevalence of cutaneous and adipose tissue neoplasms in patients with MEN1 having a comprehensive dermatologic evaluation.

Study (REF)	Number of patients	Angiofibroma (%)	Collagenoma (%)	Any angiofibroma or collagenoma (%)	Melanoma (%)	Lipoma (%)	Study populations
Darling et al. (13)	32	28 (88%)	23 (72%)	29 (91%)	Not available	11 (34%)	•17 different kindreds •Ages 14-71 years
Sakurai et al. (14)	28	12 (43%)	Not available	Not available	Not available	Not available	•14 different kindreds •Ages 21-82 years •Only face and neck were examined
Asgharian et al. (15)	48	31 (65%)	30 (63%)	39 (81%)	2 (4%)	8 (17%)	•MEN1 patients with gastrinoma •Multiple kindreds •Ages 20-77 years
Vidal et al. (16)	9	2 (22%)	0 (0%)	2 (22%)	1 (11%)	3 (33%)	•Two kindreds •Mean age 43.4 NA, Not available.years

NA, Not available.

Similar to the defining MEN1 “3Ps”, these dermatologic neoplasms have also been shown to have LOH with allelic deletion of the *MEN1* gene (27, 28), a finding not seen in a melanocytic nevus or acrochordon from MEN1 patients (27), in an angiofibroma from a patient with tuberous sclerosis (27), or in 19 sporadic angiofibromas (29), although two of the sporadic tumors were identified to have somatic missense *MEN1* mutations.

The identification of these neoplasms in someone with an MEN1-associated endocrine tumor can help to detect a patient with MEN1 (15, 30). In the study of patients with gastrinoma (with and without MEN1), the presence of > three angiofibromas and any collagenoma had high sensitivity (75%) and specificity (95%) for identifying patients with MEN1 (15). Notably, MEN4, which occurs secondary to mutations in *CDKN1B*, has not been associated with cutaneous neoplasms (31) despite other phenotypic overlap with MEN1.

### Angiofibroma

Angiofibromas (**Figure 1**) are benign fibroblastic tumors that histologically comprise bland spindle cells in a variably myxoid to collagenous stroma with a prominent vascular network. They present as telangiectatic, skin colored to pink to red-brown, dome-shaped papules with a glistening surface that primarily occur in the central part of the face, especially the nose. They may be mistaken for acne but do not spontaneously resolve. Facial angiofibromas may also resemble a wide variety of other skin disorders, including rosacea, multiple trichoepitheliomas, eruptive generalized keratoacanthomas (Grzybowski type), and Muir-Torre syndrome (32). MEN1-related angiofibromas are true neoplasms that arise from cells with a mesenchymal immunophenotype that are concentrated in a perivascular location (28).

The prevalence of angiofibromas in comprehensively screened MEN1 patients ranges from 22-88% depending on the population (13–16). At the NIH (13, 15), 65-88% of patients were affected, which may reflect the likely more severe MEN1 cases that were referred to their center, whereas the lower prevalence (43%) in Japan (14) may relate to ethnicity and the low rate (22%) in Spain (16) might be a reflection of only nine patients from two kindreds being studied. Notably, in all studies, facial angiofibromas were more prevalent in MEN1 patients compared with their control populations. In MEN1 patients with any angiofibroma, multiple lesions are identified in most patients: 33% have ≥ three (14), 77% have > three (15), and 57% have > four angiofibromas (13). Generally ranging in size from 1-4 mm, angiofibromas can less frequently number >10 and even up to 50 (13, 14).

Besides MEN1, the genodermatoses associated with facial angiofibromas include the tuberous sclerosis complex (TSC), in which facial angiofibromas (formerly called adenoma sebaceum) is a defining clinical feature, and the Birt-Hogg-Dubé Syndrome [See GeneReviews^®^ (33)]. MEN1-related angiofibromas differ from those seen in TSC, in which the lesions are larger, more numerous, and earlier in onset; in TSC, the angiofibromas are also predominantly in a malar distribution and do not appear on the upper lip and its vermilion border as can be seen in MEN1 patients (13, 34). Angiofibromas are also not pathognomonic for MEN1 or TSC and can occur sporadically (35).

An angiofibroma diagnosis is usually made clinically in a patient with known MEN1 but a biopsy can facilitate the diagnosis in someone not known to be affected. Treatment may be desired for cosmetic reasons and therapeutic approaches would be similar to patients with TSC (32).

### Collagenoma

**Figure 2:**
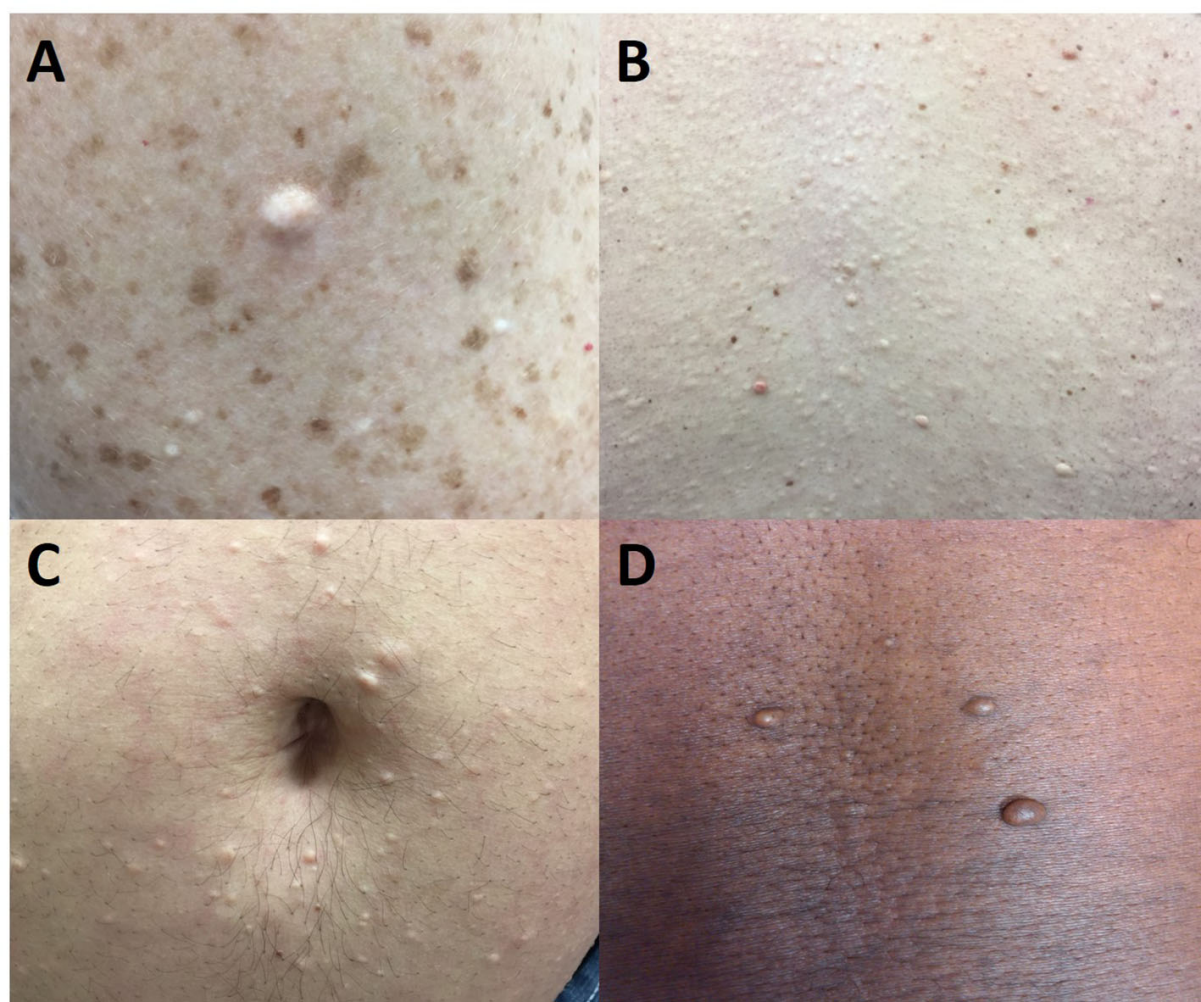
Multiple endocrine neoplasia type 1-associated collagenomas, which are benign connective tissue nevi with a dominant collagen component. They are hypopigmented or skin-colored and usually found on the neck, shoulder, and trunk. **(A)** 46-year-old woman with multiple collagenomas in addition to pigmented nevi on the arm. **(B)** 46-year-old man with innumerable collagenomas on the back. **(C)** 33-year-old man with multiple peri-umbilical collagenomas. **(D)** 46-year-old man with truncal collagenomas.

**Figure 3:**
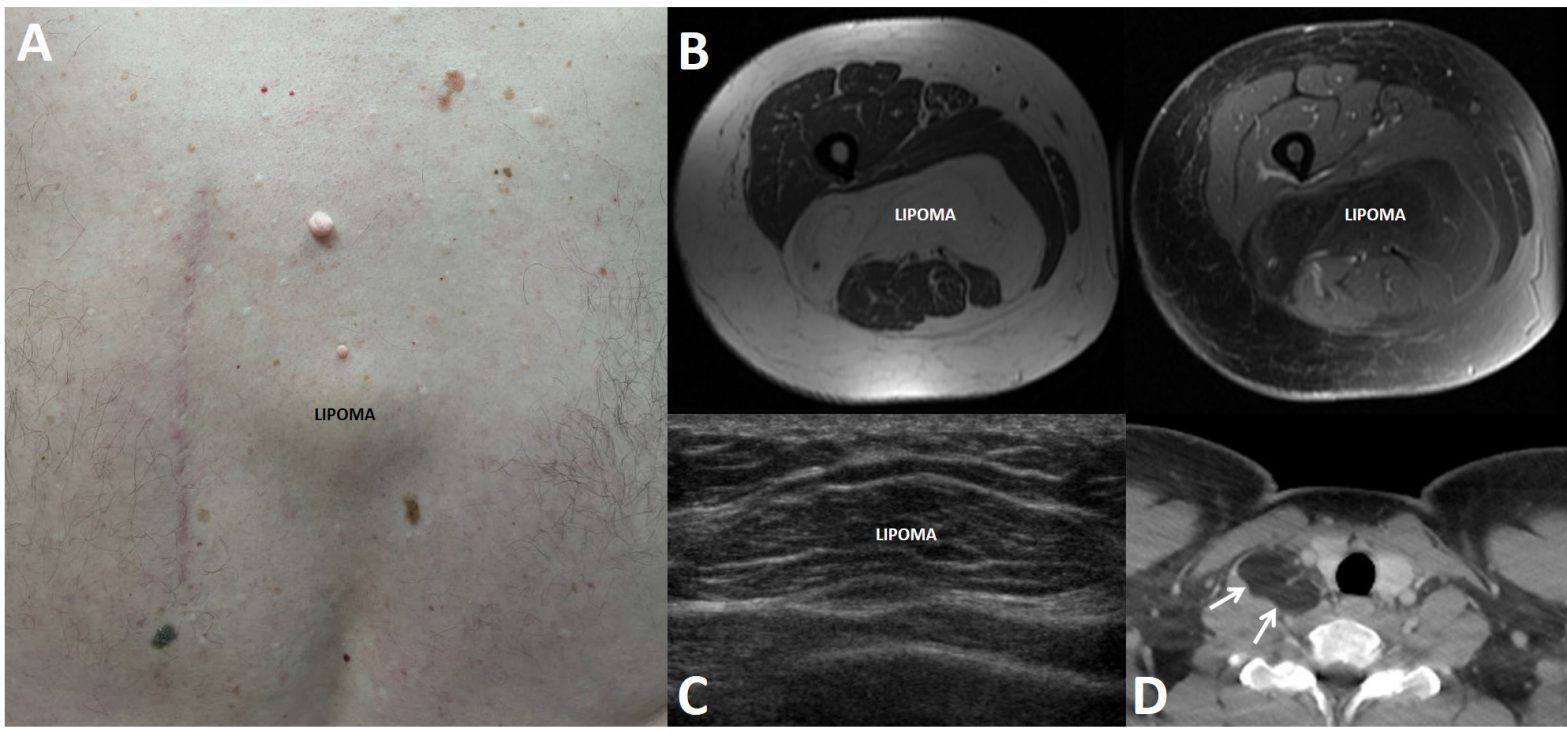
Lipomas in patients with multiple endocrine neoplasia type 1. Lipomas are benign tumors made of mature adipocytes that can arise from anywhere that fat is located. **(A)** 62-year-old man with a subcutaneous lipoma located over the spine in the mid back. Also seen is an adjacent surgical scar related to the prior resection of a large lipoma and scattered collagenomas. **(B)** 49-year-old woman with multifocal intramuscular lipomas, including a large posterior thigh lipoma as seen on axial T1-weighted pre- (left panel) and post-contrast (right) magnetic resonance imaging. **(C)** 16-year-old boy with a mobile soft tissue mass arising over the lower anterior chest. Ultrasound showed a 3.7 cm lesion consistent with lipoma. **(D)** 25-year-old man with a 3.6 cm lipoma (arrows) in the right scalene muscle, adjacent to the right thyroid lobe, as seen on contrast-enhanced computed tomography.

Collagenomas (**Figures 2**, **3**) are benign connective tissue nevi, which are hamartomas of the dermis, that have a dominant collagen component (36). Histologically, these lesions appear as unencapsulated areas of dense, thick collagen bundles arranged in a haphazard array within the reticular dermis below a region of normal-appearing papillary dermis (13, 37). They present as well-circumscribed, raised (and sometimes pedunculated), round to oval dome-shaped papules that are hypopigmented or skin-colored. They are usually subcentimeter in size (37) but can be larger (17) and are usually found on the neck, shoulders, and trunk (13); however, they have also been found on the face (38). In addition to MEN1, the genodermatoses associated with collagenomas include familial cutaneous collagenoma, Birt-Hogg-Dubé Syndrome, Buschke-Ollendorff syndrome, proteus syndrome, and TSC, in which the collagenoma is known as a shagreen patch (33, 36). The diagnosis of collagenoma is primarily a clinical one and, if treatment is required, surgery or intralesional glucocorticoids can be considered (39).

The prevalence of collagenomas in larger studies of comprehensively screened MEN1 patients ranges from 63-72% (13, 15) whereas one small study of nine patients from two Spanish kindreds did not identify any (16). In the NIH studies, these lesions were clearly more common in MEN1 patients compared with their control populations. In a more recent report from India comprised of 18 MEN1 patients from 14 unrelated families, the prevalence of collagenomas was 28% (40); however, these patients were not systematically examined by a dermatologist and the true prevalence may have been underestimated. Similar to MEN1-associated angiofibromas, collagenomas are usually multiple with 83% of MEN1 patients diagnosed with a collagenoma having at least three or four lesions (13, 15).

### Melanoma

Several publications have reported the diagnosis of melanoma in MEN1 patients. The earliest reports were single cases included in larger MEN1 series (41–44). In 2000, Nord et al. described seven cases of melanoma in unrelated MEN1 patients, including two patients with an unknown primary site of melanoma who died of disseminated disease (45). Two of the four studies primarily focusing on the dermatologic manifestations of MEN1(**Table 1**) reported patients with melanoma: 2/48 (4%) in an NIH study (15) and 1/9 (11%) in the small Spanish study (16). Another study from the NIH in 2004 described the characteristics of 107 MEN1 patients with Zollinger-Ellison syndrome. Melanoma was found in three patients (3%), but in a concomitant literature review of 1009 cases of MEN1 and Zollinger-Ellison syndrome, there were no melanomas found (46). Subsequently, two additional case reports were published (47, 48). Melanoma has been reported as a cause of death in some studies (44, 45, 49) whereas other publications studying mortality in MEN1 are notable for no deaths from melanoma (50–52).

The *MEN1* gene has not been definitively implicated in melanoma pathogenesis. Böni et al. analyzed 23 primary sporadic cutaneous melanomas and 17 metastases for *MEN1* mutations and for LOH using polymorphic markers closely linked to the *MEN1* gene (53). None of the tumors demonstrated LOH at the *MEN1* locus or a pathogenic *MEN1* variant. Nord et al. subsequently published a study including 39 sporadic melanomas, 13 melanoma cell lines, and melanomas from 20 unrelated familial melanoma kindreds that did not have germline mutations in the hereditary melanoma genes *CDKN2A* and *CDK4 (* 45). Only one somatic MEN1 mutation was identified in a sporadic tumor whereas LOH including the *MEN1* gene locus was found in 4/19 (21%) sporadic melanomas. No somatic mutations were identified in the cell lines or in the familial cases. Unfortunately, in this study, the authors were unable to test for LOH in the melanomas from MEN1 patients. In one later case report of melanoma in an MEN1 patient, LOH was not found using polymorphic DNA markers that map to the *MEN1* gene locus (47). Therefore, there are insufficient data looking for somatic alterations in the *MEN1* gene in melanomas removed from MEN1 patients. More recently, there has been some published evidence to suggest that *MEN1* may suppress the malignant phenotype of melanoma cells (54) and act as a melanoma tumor suppressor (55). However, in whole exome sequencing of 331 cutaneous melanoma patients studied in The Cancer Genome Atlas program, *MEN1* variants were not reported (56).

Cutaneous melanoma is a relatively common malignancy that is increasing in frequency and will be diagnosed in about 2% of people during their lifetimes in the United States (57). The major risk factors are being a non-Hispanic White and exposure to ultraviolet radiation. Given the very few reports in MEN1 (that don’t appear to exceed the prevalence of the general population) and the lack of supporting data that the *MEN1* gene directly plays a role in melanoma pathogenesis, it appears that melanoma diagnosed in an MEN1 patient is more likely to be coincidence.

### Summary of cutaneous tumors

There are high quality data to support that angiofibromas and collagenomas are causally related to alterations affecting the *MEN1* gene, but cutaneous melanoma should not be considered part of the MEN1 clinical spectrum. Angiofibromas/collagenomas can rarely be an initial manifestation of MEN1 and identifying these lesions in a patient presenting with another MEN1-defining tumor or having a strong family history of MEN1 can help to secure the clinical diagnosis. Most likely to be multiple in MEN1 patients and with no clear genotype-phenotype correlations, angiofibromas and collagenomas are primarily identified in adults. In most cases, these lesions do not require intervention, although some patients may want to seek treatment for cosmetic reasons. The initial and ongoing evaluation of an MEN1 patient should include a comprehensive skin examination. Patients should be educated about any skin manifestations they may have and be encouraged to practice preventative skin care and to seek consultation with a dermatologist if there is any visually concerning or symptomatic lesion.

## Lipomas and hibernomas

Lipomas, benign mesenchymal tumors made of mature adipocytes, can arise from anywhere that fat is located. They are either solitary or multiple and present as slow-growing, soft, mobile, and painless masses. In addition to occurring sporadically in the general population, lipomas can arise in various genetic disorders, including MEN1, encephalocraniocutaneous lipomatosis, *KCNK9* Imprinting Syndrome, *PTEN* Hamartoma Tumor Syndrome, *PIK3CA*-Related Overgrowth Spectrum, Birt-Hogg-Dubé Syndrome, and Myoclonic Epilepsy Associated with Ragged Red Fibers [See GeneReviews^®^ (33)].

In 1964, lipomas were first proposed by Ballard et al. (58) to be a component of MEN1, then called multiple endocrine adenomatosis, based on their finding of multicentric lipomas in 11 patients in addition to at least six previously published clinical MEN1 cases with lipomas dating back as far as 1927. Lipomas from MEN1 patients have been shown in multiple reports to have LOH at the *MEN1* locus (8, 27, 59–62), consistent with a causal relationship with MEN1, and there are no clear genotype-phenotype correlations (24, 25). Lipomas in MEN1 patients (**Figure 3**) are not infrequently multiple and are typically subcutaneous, but they can also be found more deeply and reach large sizes that may require surgical extirpation (21, 63–66). Lipomas can also be the first clinical manifestation in MEN1 patients, diagnosed as young as 9 years (24, 67), but there is not a clear correlation with patient age or disease duration (15). A single case of liposarcoma has been reported (68).

The penetrance by age 40 of lipomas in MEN1 patients has previously been estimated to 30% (26). However, like angiofibromas and collagenomas, the exact prevalence is difficult to ascertain due to the lack of large studies of MEN1 patients undergoing a comprehensive evaluation. In the literature, lipomas are reported to be found in 0.9-34% of MEN1 patients (13, 15, 16, 24, 25, 42, 46, 69–72). In the studies incorporating a comprehensive dermatologic evaluation of MEN1 patients (13, 15, 16) (**Table 1**), the prevalence was 17-34%. Other series from referral centers or large MEN1 databases reported a lipoma prevalence of 12-30% (24, 67, 70–74), but not all studies have shown a significant lipoma prevalence (42, 46, 69).

Hibernomas are rare, benign adipocytic tumors that have brown fat differentiation and can demonstrate uptake of ^18^F-fluorodeoxyglucose on positron emission tomography, potentially leading to a concern for sarcoma (75–78). Histologically they can be confused with atypical lipomatous tumors, but they do not recur after complete resection, nor do they metastasize (79). To date there have been seven published cases of hibernomas occurring in MEN1 patients (60, 75–78, 80–82), and these have primarily been located in the pelvic region and thigh. The association with MEN1 may not purely be coincidental given that LOH was identified in two 11q13 markers in a resected tumor from an MEN1 patient (60) and several publications have documented deletions of the *MEN1* gene in apparently sporadic hibernomas (83–85). However, these deletions are large and not limited to *MEN1*. Importantly, in the Nord et al. study (85), concomitant loss of the *AIP* gene, implicated in the syndrome of familial isolated pituitary adenomas, was also identified and a later study concluded that loss of *AIP* is responsible for the brown fat phenotype (86). Thus, it appears that a large deletion of 11q13 including both *MEN1* and *AIP* is necessary for hibernoma development.

In summary, lipomas are clearly established as an MEN1 clinical manifestation, occurring in up to one third of patients. Hibernomas may also very rarely be diagnosed in MEN1 patients. Because sporadic lipomas are not uncommon in the general population, the finding of a lipoma in a patient suspected to have MEN1 is not sensitive enough to make the diagnosis (15). Lipomas can grow quite large and may need to be resected due to symptoms or for cosmetic reasons, but around 70% of MEN1 patients with lipomas can be managed conservatively (67).

## Smooth muscle tumors

**Figure 4:**
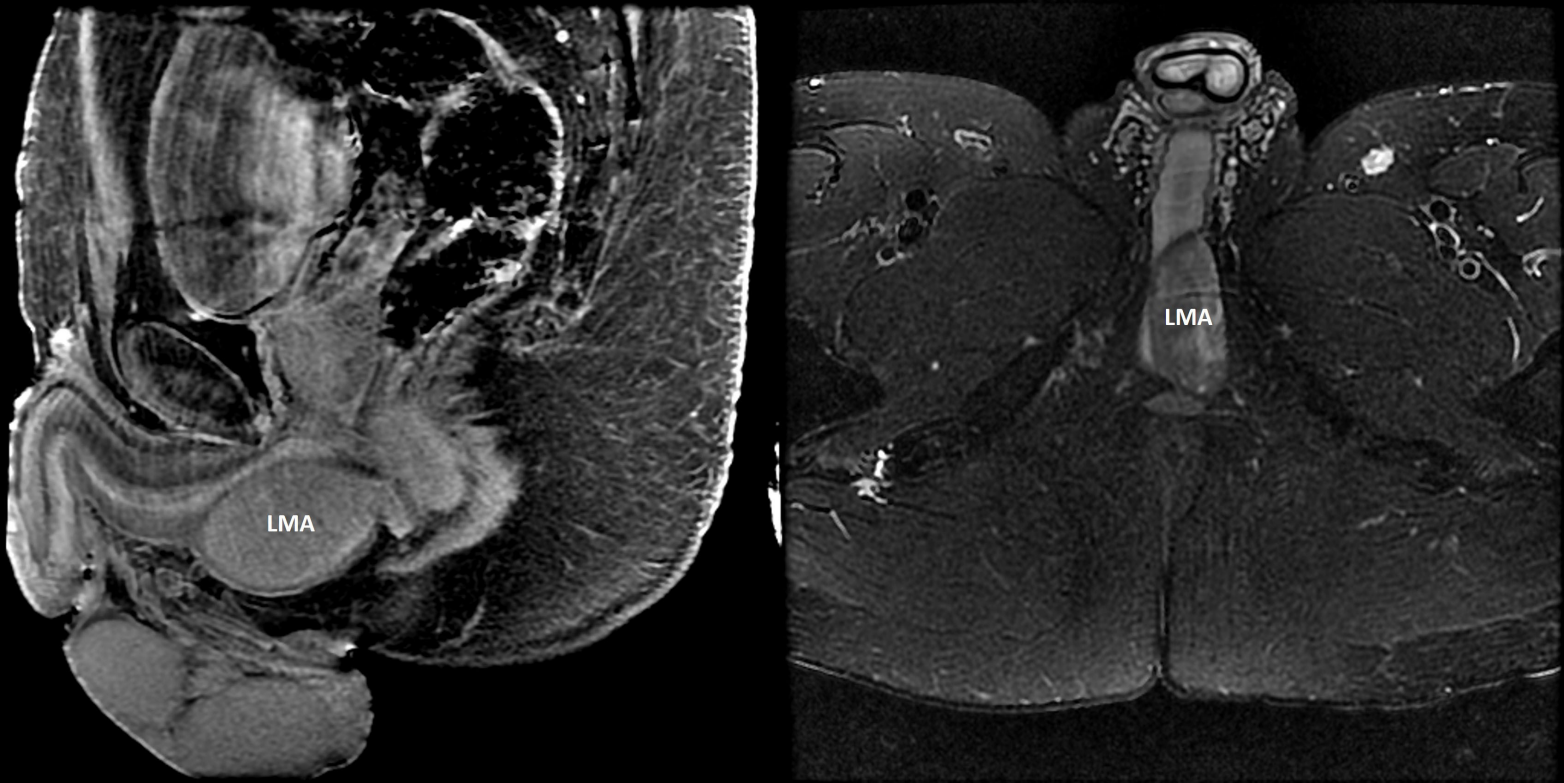
Leiomyoma (LMA). A 41-year-old man with MEN1 presented with an enlarging, painless perineal swelling. Magnetic resonance imaging (Left panel: contrast–enhanced sagittal T1-weighted fat suppressed image; right panel: axial T2-weighted fat suppressed image) identified a 5.8 x 2.6 x 3.8 cm mass in the perineum abutting the crus of the penis and the bulbous urethra. The mass was completely excised and histologic evaluation confirmed a leiomyoma. Somatic testing of the leiomyoma revealed the known germline pathogenic *MEN1* variant and evidence for biallelic loss (loss of heterozygosity) at chromosome 11q (next-generation sequencing performed by Foundation Medicine; Cambridge, Massachusetts, USA).

Smooth muscle tumors include benign leiomyomas (**Figure 4**) and their malignant counterpart, leiomyosarcoma. These mesenchymal neoplasms can arise throughout the body but most commonly affect the uterus where they are colloquially referred to as uterine fibroids. Several genetic syndromes have been associated with smooth muscle tumors, including *FH* Tumor Predisposition Syndrome (Hereditary Leiomyomatosis and Renal Cell Cancer [HLRCC]), Alport syndrome, *PTEN* Hamartoma Tumor Syndrome, MEN1, *CDC73*-Related Disorders/Hyperparathyroidism-jaw tumor syndrome, and the Birt-Hogg-Dubé Syndrome [See GeneReviews^®^ (33)]. Leiomyomas appear to occur in 10% or less of MEN1 patients (25, 26, 46, 52, 87) and 12.6% of women with MEN1 (24), but there has been no systematic evaluation of the prevalence of these tumors in MEN1 patients and so the true prevalence remains unknown, especially noting that uterine leiomyomas are very common in the general population and thus may not necessarily be an MEN1-related neoplasm in female patients.

In 1959 and 1962, necropsy reports from two men with clinical MEN1 was notable for the finding of genitourinary leiomyomata (scrotum and periprostatic) (88, 89). Subsequent early case reports of leiomyomata in patients with clinical MEN1 included a patient with an esophageal leiomyoma (58), a 39-year-old female patient with a uterine leiomyoma (90), a 43-year-old man with a pilar (cutaneous) leiomyoma derived from an arrector pili muscle (91), and a 45-year-old woman with a leiomyoma of the broad ligament (65). Ballard et al., in their 1964 publication, also reported other previously published cases of smooth muscle tumors in patients with multiple endocrine adenomatosis (58). The possible association of leiomyoma with MEN1 was first hypothesized in 1997 in two publications: a case report of a woman with clinical MEN1 and a history of uterine leiomyomatosis/leiomyosarcoma who was diagnosed with a lung lymphangioleiomyoma at age 60 years (92) and, as part of a larger study of 13 MEN1 patients, a 56-year-old female with MEN1 who had an esophageal leiomyoma (60). In the latter case, LOH at the putative *MEN1* locus was not detected using 10 polymorphic markers spanning 11q13, but the *MEN1* gene had not yet been cloned and so it was hypothesized that there could have been a small intragenic deletion or point mutation in the putative *MEN1* gene that was not detectable by conventional LOH analysis. Shortly thereafter, a second case of two distinct esophageal leiomyomata was reported in a 36-year-old woman who was ultimately diagnosed with MEN1 and confirmed to have a germline pathogenic variant (93). In both leiomyomas, there was LOH at the *MEN1* locus and the authors proposed that esophageal leiomyoma is associated with alterations in the *MEN1* gene. Additional studies further solidified the role of *MEN1* in the development of both esophageal and uterine leiomyomata (94), although LOH was not detected in four of seven leiomyomas from a single patient who had lung, esophageal and uterine tumors. Other cases of uni- and multifocal smooth muscle tumors in MEN1 patients have been reported in the English literature (24, 25, 52, 87, 95–104) and, in addition to the aforementioned sites, these tumors have arisen in the ureter (96), bladder (97), epididymis (87), ventricle (87), and small bowel (87, 103). Most cases have been in females and these neoplasms occur more rarely in men (87–89, 91, 96) (**Figure 4**).

In summary, patients with MEN1 are at risk for the development of benign and occasionally malignant (52, 92, 95) smooth muscle tumors. Females appear to be at increased risk compared with males and these tumors primarily arise in the upper gastrointestinal and genitourinary tracts. Given the primarily asymptomatic and benign nature of these tumors, routine prospective clinical screening is not warranted.

## Central nervous system tumors

### Meningioma

Meningiomas, typically slow-growing benign tumors attached to the dura mater, are believed to originate from arachnoid cells (105, 106). Exposure to ionizing radiation is a major risk factor, and patients with somatotroph pituitary adenomas also appear to have a higher risk (107). Meningiomas are located throughout the central nervous system (CNS) and symptoms relate to mass effect and are determined by tumor size and location (108). Although clinical behavior is mostly benign (WHO grade I), meningiomas can also be atypical (WHO grade II) and frankly malignant (WHO grade III). Thus, depending on their grade and location, these tumors can cause considerable patient morbidity and mortality. 

The most common intracranial tumor, meningiomas comprise 39% of all tumors and 54.5% of non-malignant primary brain and other CNS tumors; median age of diagnosis is 66 years, and the incidence of meningioma increases with age (109–111). Meningiomas are more commonly diagnosed in females and Blacks compared with males and Whites, respectively (109, 110). Meningiomas are an incidental finding in 0.9% of MRI scans in patients over age 45 years with a prevalence of 0.5% in 45- to 59-year-olds to 1.6% in persons 75 years of age or older (110). Across all ages, meningiomas are incidentally identified in 0.6% of brain MRIs, but not before age 20 years; the peak incidence is 17/1000 scans (95% CI 4–37) in 80-year-olds (111).

Various genetic syndromes have been associated with the development of meningiomas, most commonly neurofibromatosis type 2, but also the *PTEN* hamartoma tumor syndrome, Werner syndrome, *BAP1* tumor predisposition syndrome, and Rubinstein-Taybi syndrome, among others (33, 105, 106, 112). From an MEN1 perspective, initial reports of a meningioma in patients with clinical MEN1 were published in 1984 and 1996 (113, 114). In 2004, in a study of 74 eligible MEN1 patients prospectively evaluated at the National Institutes of Health (NIH), there were six cases (8.1%) of meningioma diagnosed at a mean age of 50.8 years (range 29-76) (115). All patients were asymptomatic and the meningiomas incidentally identified. One had received prior pituitary irradiation and 50% of the patients had a pituitary adenoma, but it is unknown if any of these were somatotroph adenomas. There was no meningioma growth in 60% of patients on serial imaging with a mean FU of 3.6 ± 1.8 years, but one patient ultimately died from a progressive meningioma (52). Meningioma was found late in the clinical course with a mean time of 17.6 years after the onset of MEN1. In one excised tumor, LOH was identified by all six polymorphic markers spanning the *MEN1* locus. Since the original publication from the NIH suggesting causality, there has been an additional case report of the diagnosis of meningioma in a 35-year-old woman with MEN1 and PHPT, pancreatic neuroendocrine tumor (PNET), and a somatotroph pituitary adenoma (116). No other series of MEN1 patients and meningioma has yet been published to confirm the NIH findings, although patients with meningiomas have been reported in other publications (24, 25, 43, 87, 117), including one patient who died of a meningioma (117). In the Florentine database, the prevalence of meningiomas was 2.1% (24).

Thus far there has been no strong evidence supporting a role of *MEN1* in the pathogenesis of meningiomas (118). Zhu and colleagues recently studied tumors from patients diagnosed with both pituitary adenoma and meningioma (PAM) (119, 120). In their first study of 57 PAM patients, tumors from PAM patients, compared with “sporadic” pituitary adenomas or meningiomas, had lower *MEN1* expression and, in turn, hyperactivation of the mTOR signaling pathway (119). A follow up study reported that 5/23 patients with PAM harbored a germline missense *MEN1* variant (c.1523G>A; p.G508D), but none of those patients had other MEN1 clinical manifestations and thus did not have MEN1 (120). This variant is also considered benign or likely benign by ClinVar (121).

In summary, meningiomas have been proposed as a component of MEN1 yet data are few to strongly support the role of loss of *MEN1* function in meningioma pathogenesis. The high incidence of meningioma in the general population also makes it more likely that the finding of meningioma may be coincidental, with the understanding that a patient with MEN1 might have additional risk factors for meningioma development such as growth hormone excess and a history of ionizing radiation exposure. Screening for meningioma is not recommended in MEN1 patients, but if one is identified, it should be managed similarly to patients without MEN1.

### Ependymoma

Ependymal tumors are a subtype of glioma that can occur throughout the CNS (supratentorial, infratentorial, and spinal), and they are defined by their anatomic locations and underlying molecular profiles (122–125). These tumors comprise 1.6% of all brain and other CNS tumors and are malignant in approximately 57% of all cases; proportionally they are more common (5.3%) in children < age 15 years, the group that also has the highest malignancy rate (89%) (109). Males and Whites have a higher incidence and the median age is 45 years (109); about 87% of pediatric ependymomas occur intracranially (mean age 5.0-7.8 years) whereas adult tumors more commonly (64%) occur in the spinal cord at a mean age of 45.5 years (126).

In 1991, in a study profiling the large Tasmanian MEN1 kindred, a case of spinal ependymoma was reported in a 46-year-old man, the brother of a woman with MEN1, who ultimately developed hypercalcemia and was thus diagnosed with MEN1 (65). Several years later, the first case proposing a link between MEN1 and ependymoma was published: a 51-year-old man who presented with gait disturbance and hypoesthesia was found to have a benign spinal ependymoma and was ultimately diagnosed with clinical MEN1 (PHPT, PNET, and pituitary microadenoma) (127). The authors hypothesized that there might be a common genetic etiology given the association of 11q13 translocations with ependymoma and the fact that the putative *MEN1* gene had been mapped to the same chromosomal locus. Shortly thereafter, a third case of spinal ependymoma was reported in a 29-year-old female MEN1 patient who had genetic confirmation of a pathogenic *MEN1* variant (128). Furthermore, LOH studies performed on the resected tumor showed LOH in the *MEN1* region involving the loss of the wild type alleles. The authors concluded that ependymoma is a feature of MEN1, although uncommon compared with other MEN1-related tumors. The first case of an intracranial ependymoma in an MEN1 patient was in 2010 (129). In this case, a 44-year-old woman followed for *de novo* MEN1 presented with memory loss and disorientation and was found to have a large tumor causing obstructive hydrocephalus, a tumor that ultimately led to her death. A second, well-documented case of an intracranial ependymoma arising from the cervicomedullary junction was reported in 2017 in a 33-year-old man with genetically confirmed MEN1 (130). Further testing of the tumor, which had a DNA methylation profile clustering with that of spinal ependymomas, identified somatic loss of the remaining wildtype allele due to a chromosome 11 deletion. The authors concluded that ependymoma can arise as part of MEN1 and should be potentially screened for in patients with this syndrome. Other published cases include a 53-year-old man with clinical MEN1 (no *MEN1* variant provided) and a tanycytic ependymoma arising from the filum terminale, but there was no evident abnormality at chromosome 11q13 (131). This report also referred to another case report previously published in Japanese of a 34-year-old man with a ventricular ependymoma and MEN1 (132).

The data that ependymoma may rarely arise as a component of MEN1 remain scarce and limited to seven case reports, two of which had documented LOH involving the MEN1 locus (128, 130), and generic reports of death from ependymoma in larger series of MEN1 patients (52, 133). The average age of the published cases is 41.4 years, not dissimilar from the average age of ependymoma diagnosis in the general population (109). Previous publications of familial ependymoma not associated with MEN1 (134) would suggest that there could be other not-yet-identified susceptibility genes. In addition, somatic alterations of chromosome 11 can be found in sporadic ependymomas (129, 135, 136), thus lessening the impact of finding LOH in any given tumor. Finally, given the current molecular understanding of ependymal tumors (122–125), it would be prudent to re-evaluate the published cases in this context before assigning causality to aberrations in the *MEN1* gene. Given the above, routine screening for ependymoma is not recommended in MEN1 patients but if a patient presents with new neurological signs and symptoms, appropriate imaging should be ordered to look for this very rare possibility.

## Breast carcinoma

Most recently, it has been proposed that the risk of breast carcinoma is heightened in females with MEN1. To date, there have been approximately 90 or fewer cases of breast carcinoma in women with MEN1 reported in the literature (24, 43, 44, 51, 72, 81, 87, 95, 99, 137–150); breast cancer has not yet been reported in a man with MEN1. The exact number of cases is difficult to determine due to the likely overlap of cases in the published literature and the lack of specific case numbers in one publication. **Table 2** highlights 21 individual cases in women with MEN1 for which more-detailed clinical information has been published. In these cases, the median age of diagnosis is 45 years (33-69 years), and most cases are invasive ductal carcinomas, similar to the general population. Other histologies include ductal carcinoma *in situ*, invasive lobular carcinoma, invasive micropapillary carcinoma, and lobular carcinoma *in situ*, noting that the last histology is no longer considered to be a malignant lesion (151). The vast majority of breast cancers in MEN1 females are unilateral and unifocal, and there is no consistent pattern of hormone receptor or human epidermal growth factor receptor 2 (HER2) expression, although over 80% express the estrogen receptor. Furthermore, the tumors mostly appear to be smaller and without lymph node or distant metastases. The French and Belgian *Groupe d’Etude des Tumeurs Endocrines* reported five female patients with MEN1 who died of breast cancer (51). However, the number of breast cancer deaths in their MEN1 population was similar to the estimated breast cancer mortality in the general population and thus did not *a priori* suggest a heightened risk of aggressive breast cancer in MEN1 patients. Deaths due to breast carcinoma in patients with MEN1 have also been reported in other publications (44, 52, 87, 137, 141), but the numbers are small and, on the surface, don’t appear to exceed that which might be identified in the general population without MEN1. Furthermore, other papers looking at mortality in MEN1 are notable for the lack of breast cancer cases (49, 50, 52, 117, 152–154).

**Table 2:** Clinical details of 21 cases of breast cancer in women with genetically confirmed MEN1.

Case # (REF)	Age at diagnosis	Histology	Laterality and focality	TNM Stage^a^	ER, PR, HER2 status	Somatic LOH at MEN1 locus	Comments/associated MEN1 tumors
1 (138)	55	Ductal (Invasive)	Unilateral	T1N1M0	ER+ PR- HER2-	NO	Menin expression-
2 (138)	38	Ductal (Invasive)	Unilateral	T3N1M0	ER+ PR+ HER2+	NO	Menin expression-
3 (138)	44	Ductal (Invasive)	Unilateral	T1N0M0	ER- PR- HER2-	NO	Menin expression+
4 (138)	61	Ductal (Invasive)	Unilateral	T1N1M0	ER+ PR- HER2-	NO	Menin expression-
5 (138)	52	Lobular (Invasive)	Unilateral	T1N0M0	ER+ PR+ HER2+	NO	Menin expression-
6 (138)	53	Ductal (Invasive)	Unilateral	T1N0M0	ER+ PR+ HER2-	YES	Menin expression-
7 (138)	45	Micropapillary (Invasive)	Unilateral	T1N1M0	ER+ PR- HER2-	YES	Menin expression-
8 (138)	42	Ductal (Invasive)	Bilateral	T1N0M0	ER- PR+ HER2-	YES	Menin expression-
9 (138)	33	Ductal (Invasive)	Unilateral	T1N1M0	ER+ PR+ HER2+	NO	Menin expression+
10 (138)	46	Ductal (Invasive)	Unilateral	T1N0M0	ER+ PR+ HER2-	Not available	Menin expression-
11 (99)	45	Ductal (invasive and *in situ*)	Unilateral	T1N+M0	ER+ PR+ HER2-	Not available	Polymorphism of the *RET* and *BRCA1* genes; PHPT, PRL, PNET, ACT, uterine leiomyoma, thymic NET
12 (139)	36	NA (“invasive ductal lobular carcinoma”)	Unilateral	TxN2M0	ER+ PR+ HER2-	Not available	PHPT, PRL, micronodular adrenal hyperplasia, lipoma
13 (142)	41	Ductal (Invasive)	Unilateral	T1N0M0	ER- PR- HER2-	Not available	PHPT
14 (143)	48	Ductal (*in situ*)	Unilateral; multifocal	TisN0M0	ER+ PR+ HER2-	Not available	PHPT, meningioma, ACT, gastrinoma/PNET, PRL
15 (144)	56	Ductal (Invasive)	Unilateral	T1aN0M0	ER+ PR+ HER2-	Not available	VUS in *ATM;* PNET; PRL; PHPT, ACT
16 (81)	42	Lobular (Invasive) (Multiple micro-foci of infiltrating lobular carcinoma within a hibernoma)	Bilateral (metachronous)	T3N2M0	ER+ PR- HER2-	Not available	PHPT, ACT, insulinoma; *BRCA1/2*-
17 (146)	47	Ductal (Invasive)	Unilateral	T2N0M0	ER+ PR+ HER2-	Not available	Lung NET, PHPT, PNET
18 (149)	38	Ductal (*in situ*)	Unilateral	T2N0M0	ER+ PR- HER2-	YES	insulinoma Subclonal somatic pathogenic variants in *BRCA2* and *TP53* in the breast tumor
19 (148)	69	NA (Invasive)	Unilateral	TxNxM0	ER+ PR? Her2?	Not available	*BRCA1/2* – PHPT, gastric NET (chronic atrophic gastritis)
20 (72)	36	Lobular *in situ* ^b^	Unilateral	Not available	Not available	Not available	2 patients from the same family
21 (72)	54	Lobular (Invasive)	Unilateral	Not available	Not available	Not available	2 patients from the same family

a **T**umor, Lymph **N**ode, **M**etastasis staging as provided in the published cases or as determined by the author using the AJCC 8^th^ edition (151).

b No longer considered to be a malignant neoplasm (151).

ER, estrogen receptor; PR, progesterone receptor; HER2, human epidermal growth factor receptor 2; LOH, loss of heterozygosity; +, positive; -, negative; ?, unknown; PHPT, primary hyperparathyroidism; PRL, prolactinoma; ACT, adrenocortical tumor; NET, neuroendocrine tumor; PNET, pancreatic neuroendocrine tumor; VUS, variant of unknown significance. NA, Not available.

The first postulation that the *MEN1* gene might play a role in breast cancer pathogenesis was made in 2004 by Honda et al. who reported a case of a 44-year-old woman with a parathyroid adenoma, aldosteronoma, and a scirrhous (ductal) breast carcinoma (155). The patient was found to have a common germline *MEN1* single nucleotide polymorphism (thus she did not have MEN1), and the breast and parathyroid tumors showed LOH at the *MEN1* locus.

In 2014, a study from the Netherlands reported the incidence of breast cancer from the Dutch longitudinal MEN1 database (138). In 190 female patients, 12 (6.3%) developed invasive, primarily ductal, breast carcinomas. The relative risk (RR) of breast cancer was 2.83 (p<0.001) with a standardized incidence ratio (SIR) of 2.14 (95% confidence interval [CI] 1.18-3.86). The mean age at diagnosis was 48.0 ± 8.8 years, compared with an age of 60-65 years in the general Dutch population. This observation was similar in three independent MEN1 cohorts from France (RR 2.33; p=0.03), the United States (RR 2.40; p=0.11), and Australia (Tasmania) (RR 2.31; p=0.22), although only one of these comparisons was significant, likely due to the small numbers of breast cancer patients in those MEN1 registries. Combining the three verification cohorts, the SIR was 1.96 (95% CI, 1.33 to 2.88). In total, amongst all the databases, there were 44 cases of breast cancer in 865 female MEN1 patients (5.1%) at an average age of diagnosis of 50 years. In evaluable tumors from the Dutch women with confirmed germline *MEN1* mutations, LOH at the *MEN1* locus was demonstrated in three of nine cancers and reduction of nuclear menin expression >50% was identified in eight of ten tumors.

Interestingly, three out of the 12 Dutch breast cancer patients had a history of hyperprolactinemia. There appears to be a positive association between elevated levels of prolactin and the development of invasive breast cancer (RR 1.42; CI 1.24-1.60) (156). Therefore, the risk of breast cancer could theoretically be heightened inwomen with MEN1 who concomitantly have hyperprolactinemia/prolactinoma.

In 2017, a follow-up cross-sectional case control study within the Dutch cohort showed that the increased risk of breast cancer was not associated with other known risk factors (age at menarche, age at first child birth, parity, oral contraception use, obesity, breast feeding, alcohol consumption, and smoking) or familial breast cancer occurrence (140). In this study, the median age at breast cancer diagnosis in 22 women (11 genetically confirmed; 11 obligate carriers) was 45 years (range 30 to 80 years). In contrast, relatives without MEN1 and breast cancer had a median age of diagnosis of 57.5 years (range 40 to 85 years; p=0.03), closer to the mean age at breast cancer diagnosis (61.2 years) in the Dutch population. No women in this study had been diagnosed with a prolactinoma. The authors also assessed exposure to radiation from computerized tomography (CT) scans done for MEN1 surveillance and the frequency of CT scans was similar for women with and without breast cancer. In mutation-negative, clinical MEN1 patients, the age of breast carcinoma diagnosis was not different from the general Dutch population.

From a basic science perspective, there are data to suggest a role for the *MEN1* gene in hormone-dependent breast cancer (157–160). Menin is a coactivator for ERα-mediated transcription and the majority of disease-related *MEN1* mutations prevent menin-ERα interaction (161, 162). In a mouse model, *MEN1*-disrupted mammary glands are significantly more likely to develop mammary intraepithelial neoplasia (MIN) that, in most cases, display complete menin inactivation (163). In this same study, reduced menin expression was also found in a large proportion of two independent cohorts of breast carcinoma patients. There are also data to suggest that menin expression might in fact promote tamoxifen resistance: in 65 ER-positive breast cancer samples from women treated with adjuvant tamoxifen for 2–5 years, menin-positive tumors were found to have a worse relapse-free survival compared with menin-negative ones (162). An older study of 24 breast cancers did not find mutations in *MEN1* exon 2 (where most mutations had been described up to that date) (164). In The Cancer Genome Atlas study of 510 breast tumors published in 2012, mutations in *MEN1* were not reported (165). In a later study looking at the molecular landscape in 560 breast cancers, somatic *MEN1* mutations were extraordinarily rare (166). Most recently, in a large study sequencing 34 putative germline susceptibility genes in 60,466 women with breast cancer and 53,461 controls, protein-truncating (odds ratio 0.37 [95% CI 0.07–1.97; p=0.24]) and rare missense *MEN1* variants (odds ratio 0.86 [95% CI 0.66–1.12; p=0.25]) were not associated with an increased breast cancer risk (167).

Intensified breast cancer screening for women with MEN1 was suggested by Dreijerink et al. in their initial publication (138) but the role of enhanced screening in this population was subsequently questioned (168). In 2017, the Dutch group proposed that women with MEN1 start biennial screening at age 40, which is 10 years before the Dutch screening program that starts at age 50 years (140). As stated by Dreijerink et al. in a letter to the editor (169), the decision to intensify breast cancer screening in MEN1 patients should not be taken lightly, and the dilemma is whether the benefits of detecting early stage cancers will outweigh the potential harms from the surveillance (i.e. earlier mammography and its attendant increased lifetime exposure to radiation) and the risk of false positives that can lead to unnecessary procedures, patient anxiety, and increased health care costs.

Although the data derived from the Dutch MEN1 cohort are compelling, causality has not yet been unequivocally demonstrated (168). First, LOH affecting chromosome 11q occurs in sporadic breast cancer (170, 171) and thus the finding of LOH in the published cases may be incidental. As suggested by Brennan (168), more extensive LOH studies are required to determine the extent of chromosome 11q loss in breast tumors from MEN1 patients compared with matched, sporadic breast cancer controls. Second, the rare possibility of patients coincidentally having pathogenic germline variants in other breast cancer susceptibility genes needs to be considered (172, 173). Third, in more recent population studies, germline *MEN1* variants are not clearly associated with a higher risk of breast cancer (167), and other publications have not reported a breast cancer risk in MEN1 patients greater than that of the general population (51, 145). Fourth, for a tumor syndrome classically associated with multifocality, it is striking that there are not more multifocal or bilateral breast cancers in the published cases, not to mention the lack of male breast cancer, the risk of which should theoretically also be increased. Fifth, although the onset of breast cancer in Dutch MEN1 patients is approximately 15 years earlier than the general population (140), the overall point prevalence of breast cancer [5.1% in 865 women from four databases (138); 4.2% in the Florentine database (24)] may not be higher than the lifetime risk of breast cancer in any woman. In the United States, for example, a woman has a 12.9% probability (1 in 8) of developing invasive breast cancer from birth to death (57). Sixth, prior studies from Sweden identified a significant association between PHPT and subsequent incidence of breast cancer in women (174, 175) and another case series found an association of presumed familial isolated PHPT and breast cancer (176). There may also be an association between insulinoma diagnosis and breast cancer risk (150). Therefore, a link between breast cancer and MEN1 may not necessarily be directly related to a pathogenic *MEN1* variant but rather to an MEN1 clinical manifestation such as prolactinoma, hyperparathyroidism, or insulinoma. Finally, the earlier age of diagnosis may be more related to a surveillance bias (via routine chest imaging for neuroendocrine tumor surveillance) rather than more aggressive biology. Further understanding of how these patients came to be diagnosed with breast cancer would be important.

In summary, it has recently been suggested that the risk of breast carcinoma is increased in women with MEN1, but further studies are required to prove causality with more certainty. If breast cancer is indeed part of the MEN1 tumor spectrum, it appears to be one with a relatively low clinical penetrance and without a clearly heightened risk of death from metastatic disease. Women with MEN1 should be counseled about this possible increased risk and be encouraged to be “breast aware” and perform routine breast self-examination. Whether or not formal screening mammography should commence earlier than the general population remains an area of uncertainty, and the age at which to begin screening should also consider a woman’s unique familial and personal risk factors for breast carcinoma.

## Conclusion

MEN1 is a rare, autosomal-dominantly inherited tumor syndrome classically defined by tumors arising from the “3 Ps”: **P**arathyroids, **P**ituitary, and the endocrine **P**ancreas. From its earliest descriptions, MEN1 has been associated with other endocrine and non-endocrine neoplastic manifestations, and the data strongly support an association with neoplasms of the skin (angiofibromas and collagenomas), adipose tissue (lipomas and hibernomas), and smooth muscle (leiomyomas). Although CNS tumors, melanoma, and, most recently, breast cancer have been reported as MEN1 clinical manifestations, the published evidence to date is not yet of sufficient high quality to include these tumors in the MEN1 clinical spectrum. Well-designed, multicenter studies will help us to understand better the relationship of these tumors to MEN1, in addition to verifying the true prevalence and penetrance of the well-documented neoplastic associations. Nevertheless, patients affected by MEN1 should be aware of these non-endocrine manifestations and providers should be encouraged always to think beyond the “3 Ps” when treating an MEN1 patient.

## Author contributions

The author confirms being the sole contributor of this work and has approved it for publication.

## Acknowledgments

The author sincerely thanks the patients and families with MEN1 who have taught him so much and who continue to do so at each encounter. The author is also indebted to Maria Luisa Brandi, Rajesh Thakker, and Gerlof Valk for their review of the manuscript.

## Conflict of interest

The author declares that the research was conducted in the absence of any commercial or financial relationships that could be construed as a potential conflict of interest.

## Publisher’s note

All claims expressed in this article are solely those of the authors and do not necessarily represent those of their affiliated organizations, or those of the publisher, the editors and the reviewers. Any product that may be evaluated in this article, or claim that may be made by its manufacturer, is not guaranteed or endorsed by the publisher.
